# Radiographically detected pulp stones on panoramic radiographs and their association with systemic diseases: a retrospective comparative cross-sectional study

**DOI:** 10.1186/s12903-026-08983-3

**Published:** 2026-06-23

**Authors:** Yakup Şen, Sümeyye Coşgun Baybars

**Affiliations:** Department of Oral and Maxillofacial Radiology, Faculty of Dentistry, Fırat University, Elazığ, Türkiye

**Keywords:** Dental pulp calcification, Panoramic radiography, Diabetes mellitus, Cardiovascular disease, Systemic diseases

## Abstract

**Objective:**

The aim of this retrospective comparative cross-sectional study was to evaluate the prevalence of radiographically detected pulp stones on panoramic radiographs and investigate their possible association with systemic diseases.

**Methods:**

A total of 1,000 panoramic radiographs obtained from the archive of the Faculty of Dentistry at Fırat University between 2019 and 2025 were retrospectively analyzed. Participants were divided into four groups: control (*n* = 400), cardiovascular disease (*n* = 200), diabetes mellitus (DM) (*n* = 200), and kidney stone (*n* = 200). The presence of radiographically detected pulp stones, tooth location (according to the FDI system), and age groups were recorded. Statistical analyses included the chi-square test and age- and sex-adjusted binary logistic regression analyses. Inter- and intra-observer agreement was assessed using Cohen’s kappa coefficient.

**Results:**

The overall prevalence of radiographically detected pulp stones was 28.1%. The prevalence was significantly higher in the diabetes mellitus (38.5%), cardiovascular disease (34.0%), and kidney stone (31.0%) groups compared with the control group (18.5%) (*p* < 0.001). In the unadjusted analysis, the odds of pulp stone presence were significantly higher in the diabetes mellitus (OR = 2.25), cardiovascular disease (OR = 1.89), and kidney stone groups (OR = 1.62). However, these crude associations changed substantially after adjustment for age and sex, with adjusted estimates moving below the null value, suggesting substantial confounding by age and limited comparability between the disease and control groups. Age remained the strongest independent predictor in the adjusted model (*p* < 0.001). The highest prevalence was observed in the 46–60 age group (41.0%), and females showed a significantly higher prevalence than males (*p* = 0.04). Radiographically detected pulp stones were most frequently observed in the maxillary and mandibular first molars.

**Conclusion:**

Radiographically detected pulp stones were more frequently observed in individuals with systemic diseases, particularly diabetes mellitus and cardiovascular disease. However, although higher crude prevalence rates were observed in the systemic disease groups, these associations changed substantially after adjustment for age and sex, with adjusted estimates moving below the null value, suggesting substantial confounding by age and limited comparability between the disease and control groups. Due to the retrospective cross-sectional design and the limitations of panoramic radiography, the results should be interpreted cautiously. Further prospective multicenter studies using advanced imaging modalities are needed to clarify these associations.

## Introduction

Pulp stones are nodular calcifications that develop within the dental pulp tissue [1]. Morphologically, they can be classified as free, attached to dentin, or embedded within the dentin as diffuse calcifications [2]. Radiographically, they appear as radiopaque structures within the pulp chamber or root canal and may occasionally occupy a substantial portion of the pulp space. Although these structures are typically a few millimeters in diameter, they are more readily detected when their size exceeds 2–3 mm [3, 4].

The exact etiopathogenesis of pulp stones remains unclear. However, several factors have been implicated, including pulpal degeneration, chronic local irritation, dental caries, orthodontic forces, periodontal disease, aging, vascular disturbances, systemic conditions, and genetic predisposition [5, 6]. In some cases, their occurrence in the absence of any identifiable cause further supports the multifactorial nature of their development.

Recent studies have suggested that pulp stones may be associated not only with local dental factors but also with certain systemic conditions. In particular, an association between pulp stones and renal calculi has been reported [7]. Moreover, cardiovascular diseases, which are among the leading causes of morbidity and mortality worldwide, have been linked to pulpal calcifications [8–10]. In addition, hyperglycemia has been suggested to induce vascular and structural alterations in the pulp tissue, potentially leading to pulpal calcifications [11]. Nevertheless, the relationship between pulp stones and systemic diseases remains inconclusive, with conflicting findings reported across different populations.

Furthermore, epidemiological studies evaluating the prevalence of pulp stones in relation to demographic and clinical variables such as age, sex, and tooth type remain limited. In particular, studies comprehensively assessing these parameters together with systemic diseases are scarce, highlighting the need for further investigation. Although several previous studies have investigated the prevalence of pulp stones, few have simultaneously evaluated multiple systemic disease groups within the same study population. Furthermore, data regarding the possible association between pulp stones and systemic diseases in Turkish populations remain limited. Therefore, additional epidemiological evidence is needed to better clarify these associations. To the best of our knowledge, few previous studies have comprehensively evaluated multiple systemic disease groups together with demographic and tooth-related variables in a large Turkish population using panoramic radiographs. Unlike many previous prevalence studies, the present study also assessed the effect of age- and sex-adjustment on the observed associations between radiographically detected pulp stones and systemic diseases, emphasizing the potential confounding effect of age.

Therefore, the aim of the present study was to evaluate the prevalence of radiographically detected pulp stones according to age, sex, and tooth type, and to investigate their possible association with diabetes mellitus, cardiovascular disease, and kidney stones compared with healthy controls using panoramic radiographs.

## Materials and methods

This retrospective comparative cross-sectional study was conducted using panoramic radiographs obtained from the archive of the Department of Oral and Maxillofacial Radiology, Faculty of Dentistry, Fırat University, between 2019 and 2025. The study protocol was approved by the Non-Interventional Research Ethics Committee of Fırat University (Date: 30.12.2025; Approval No: 42894). The study was conducted in accordance with the Declaration of Helsinki. Due to the retrospective nature of the study, the requirement for informed consent was waived by the Ethics Committee.

Eligible panoramic radiographs were retrospectively identified from archived records according to predefined comparison groups. Within each disease category, eligible records were consecutively selected until the target group size was reached. The control group was selected from individuals without documented systemic disease during the same period. A total of 1,000 panoramic radiographs were included in the final analysis. Participants were divided into four groups: control group (healthy individuals, *n* = 400), cardiovascular disease group (*n* = 200), diabetes mellitus (DM) group (*n* = 200), and kidney stone group (*n* = 200). Systemic conditions were determined based on patients’ clinical records. The cardiovascular disease group included patients diagnosed with hypertension, coronary artery disease, and other cardiovascular conditions. Due to the heterogeneous nature of this group, systemic diagnoses were determined according to documented medical records.

Tooth-level findings were presented descriptively to demonstrate anatomical distribution patterns rather than independent risk analyses.

Due to the retrospective design of the study, the diagnosis was based solely on panoramic radiographic appearance, and no CBCT or histopathological confirmation was available. Therefore, radiographically detected pulp stones were defined as discrete radiopaque masses located within the pulp chamber or root canal space on panoramic radiographs.

Inclusion criteria were individuals aged 18 years or older with diagnostically acceptable panoramic radiographs and complete medical records. Exclusion criteria included poor image quality, severe artifacts caused by orthodontic appliances or prostheses, and incomplete clinical data. Teeth with restorative artifacts preventing reliable radiographic assessment were excluded at the tooth level rather than excluding the entire patient radiograph. Patients presenting with multiple systemic diseases were excluded from disease-specific comparison groups to preserve group exclusivity and reduce overlapping confounding effects.

All panoramic radiographs were obtained using a Planmeca ProMax device (Planmeca OY, Helsinki, Finland) with standardized exposure parameters (10 mA, 85 kVp, 14 s). The images were evaluated on a high-resolution monitor in a dimly lit room by two experienced oral and maxillofacial radiologists who were blinded to the study groups during radiographic evaluation.

**Fig. 1:**
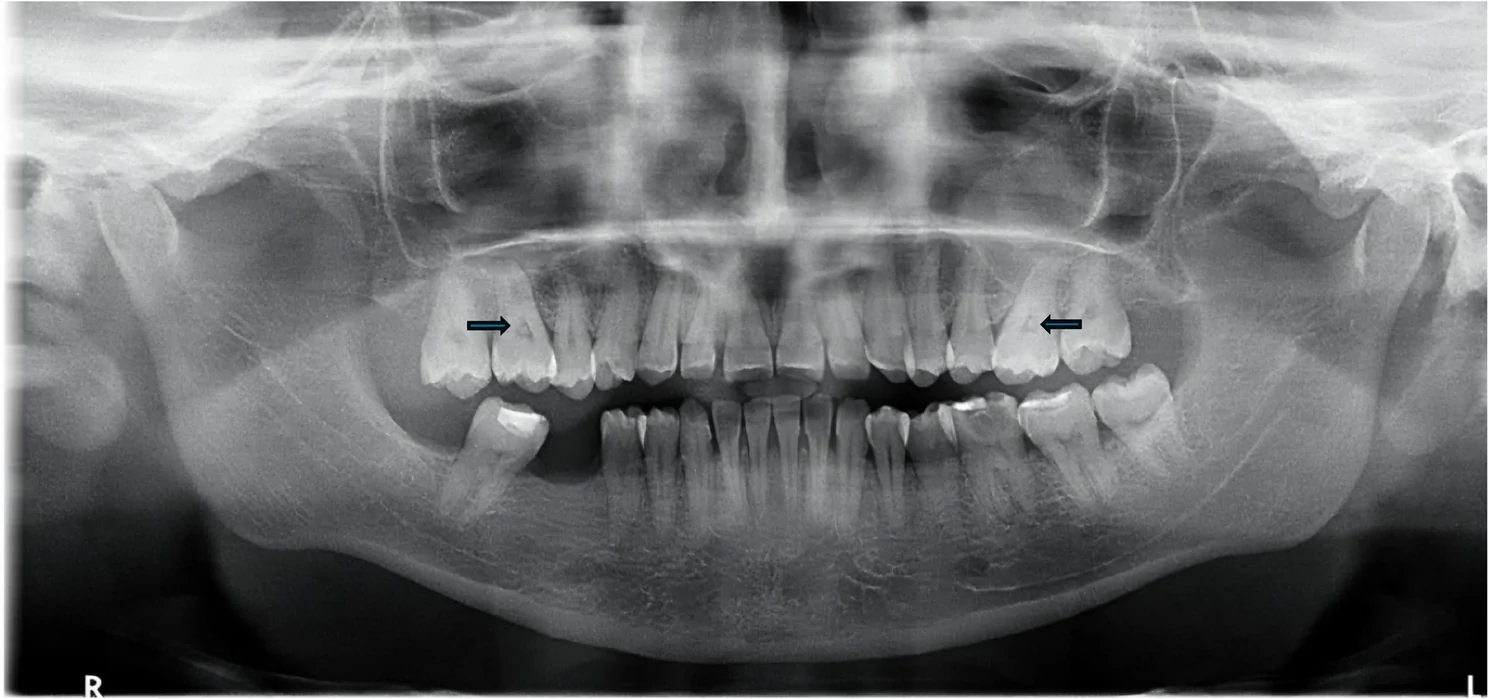
A panoramic radiograph of an individual from the cardiovascular disease group demonstrates radiopaque structures consistent with radiographically detected pulp stones within the pulp chambers of teeth 16 and 26

The presence or absence of radiographically detected pulp stones, tooth location (according to the FDI system), and age groups (18–30, 31–45, 46–60, and > 60 years) were recorded (Fig. 1).

Before the main evaluation, the observers jointly reviewed representative radiographs to standardize the diagnostic criteria. In cases of disagreement, a consensus evaluation was performed. To assess intra-observer reliability, 100 radiographs were re-evaluated after a two-week interval. Inter-observer agreement was assessed by comparing 200 radiographs between the two observers. Agreement levels were calculated using Cohen’s kappa coefficient. During observer agreement analysis, localization was categorized according to tooth groups and jaw localization.

### Statistical analysis

Statistical analyses were performed using IBM SPSS Statistics version 22.0 (IBM Corp., Armonk, NY, USA). Descriptive statistics were presented as frequencies and percentages for categorical variables and as mean ± standard deviation for continuous variables. The chi-square test was used to compare categorical variables between groups.

Binary logistic regression analyses were performed to evaluate the association between systemic diseases and the presence of radiographically detected pulp stones. Regression analyses evaluating systemic associations were conducted at the patient level. The dependent variable was the presence of radiographically detected pulp stones (0 = absent, 1 = present). Independent variables included diabetes mellitus, cardiovascular disease, and kidney stones, with the control group used as the reference category.

Both unadjusted (crude) odds ratios (ORs) and multivariable adjusted odds ratios (aORs) with 95% confidence intervals (95% CIs) were calculated. Age and sex were included as covariates in the adjusted regression model to control for potential confounding effects. The results were reported as ORs, aORs, 95% CIs, and exact p-values. A p-value < 0.05 was considered statistically significant.

## Results

A total of 1000 panoramic radiographs were evaluated, and the overall prevalence of radiographically detected pulp stones was 28.1% (*n* = 281). The prevalence was significantly higher in females (30.8%) than in males (26.2%) (*p* = 0.04), with the highest prevalence observed in the 46–60 years age group (41.0%).

Binary logistic regression analyses demonstrated higher crude odds of pulp stone presence in systemic disease groups compared with controls. In the unadjusted analysis, compared with the control group, the odds of pulp stone occurrence were higher in the diabetes mellitus (OR = 2.25), cardiovascular disease (OR = 1.89), and kidney stone groups (OR = 1.62). However, these crude associations changed substantially after adjustment for age and sex, with adjusted estimates moving below the null value, suggesting substantial confounding, particularly by age, and limited comparability between the disease and control groups.

Regarding tooth distribution, radiographically detected pulp stones were most frequently observed in the maxillary first molars (24.5%) and mandibular first molars (20.9%). Among the systemic disease groups, the highest prevalence was found in the diabetes mellitus group (38.5%), followed by the cardiovascular disease group (34.0%) and the kidney stone group (31.0%), whereas the control group showed the lowest prevalence (18.5%) (*p* < 0.001).

Significant differences were observed among the groups regarding age and pulp stone prevalence (*p* < 0.001). However, sex distribution did not significantly differ between the groups (*p* = 0.81) (Table 1).

**Table 1:** Demographic Characteristics and Distribution of Pulp Stones Among Study Groups

Variable	Control (*n* = 400)	Cardiovascular Disease (*n* = 200)	Diabetes Mellitus (*n* = 200)	Kidney Stones (*n* = 200)	*p*-value
Mean age ± SD	30.2 ± 8.4	58.4 ± 11.2	61.1 ± 10.5	56.9 ± 9.8	< 0.001
Female, I (%)	220 (55.0)	112 (56.0)	118 (59.0)	109 (54.5)	0.81
Male, *n* (%)	180 (45.0)	88 (44.0)	82 (41.0)	91 (45.5)	
Patients with pulp stones, n (%)	74 (18.5)	68 (34.0)	77 (38.5)	62 (31.0)	< 0.001

Observer reliability was high, with intra-observer kappa values ranging from 0.84 to 0.89 and inter-observer kappa values ranging from 0.81 to 0.87, indicating good to excellent agreement and reproducibility (Table 2).

**Table 2:** Inter- and intra-observer agreement based on kappa statistics

Evaluation	Kappa (κ)	Level of Agreement
Intra-observer (presence/absence)	0.89	Very good
Intra-observer (localization)	0.84	Good
Inter-observer (presence/absence)	0.87	Very good
Inter-observer (localization)	0.81	Good

In the unadjusted analysis, all systemic disease groups demonstrated significantly higher odds of pulp stone presence compared with the control group (Table 3).

**Table 3:** Unadjusted Logistic Regression Analysis for Pulp Stone Presence

Variable	Crude OR	95% CI	*p*-value
Cardiovascular disease	1.89	1.30–2.76	0.001
Diabetes mellitus	2.25	1.55–3.26	< 0.001
Kidney stones	1.62	1.10–2.37	0.014

In the multivariable logistic regression analysis adjusted for age and sex, age remained the strongest independent predictor of pulp stone presence (aOR = 1.05, 95% CI: 1.04–1.07, *p* < 0.001). Female sex was also associated with increased odds of pulp stones. Although systemic disease groups showed higher crude odds ratios, the adjusted analyses demonstrated substantial changes in these associations, with adjusted estimates moving below the null value (OR = 1) after controlling for age and sex. These findings suggest substantial confounding, particularly by age, and limited comparability between the disease and control groups (Table 4).

**Table 4:** Multivariable Logistic Regression Analysis Adjusted for Age and Sex

Variable	Adjusted OR (aOR)	95% CI	*p*-value
Cardiovascular disease	0.47	0.27–0.80	0.005
Diabetes mellitus	0.55	0.32–0.94	0.028
Kidney stones	0.35	0.20–0.61	< 0.001
Age	1.05	1.04–1.07	< 0.001
Female sex	1.35	1.01–1.80	0.039

**Fig. 2:**
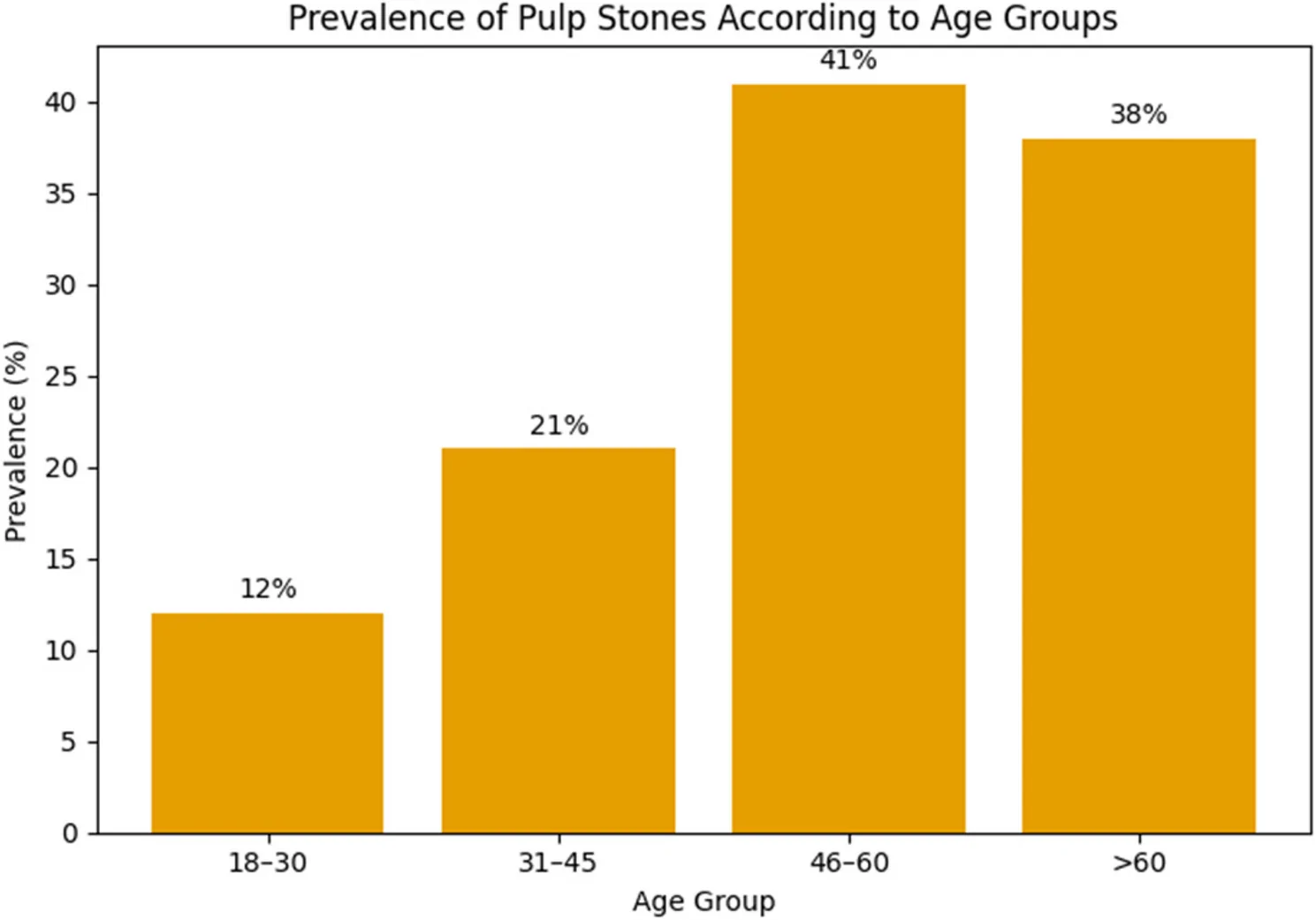
Prevalence of pulp stones according to age groups

The lowest prevalence was observed in the 18–30 years group (12%), whereas the prevalence in the 31–45 years group was 21%. The highest prevalence was found in the 46–60 years group (41%), followed by individuals older than 60 years (38%). The prevalence increased up to the 46–60 age group and slightly decreased thereafter (Fig. 2).

**Fig. 3:**
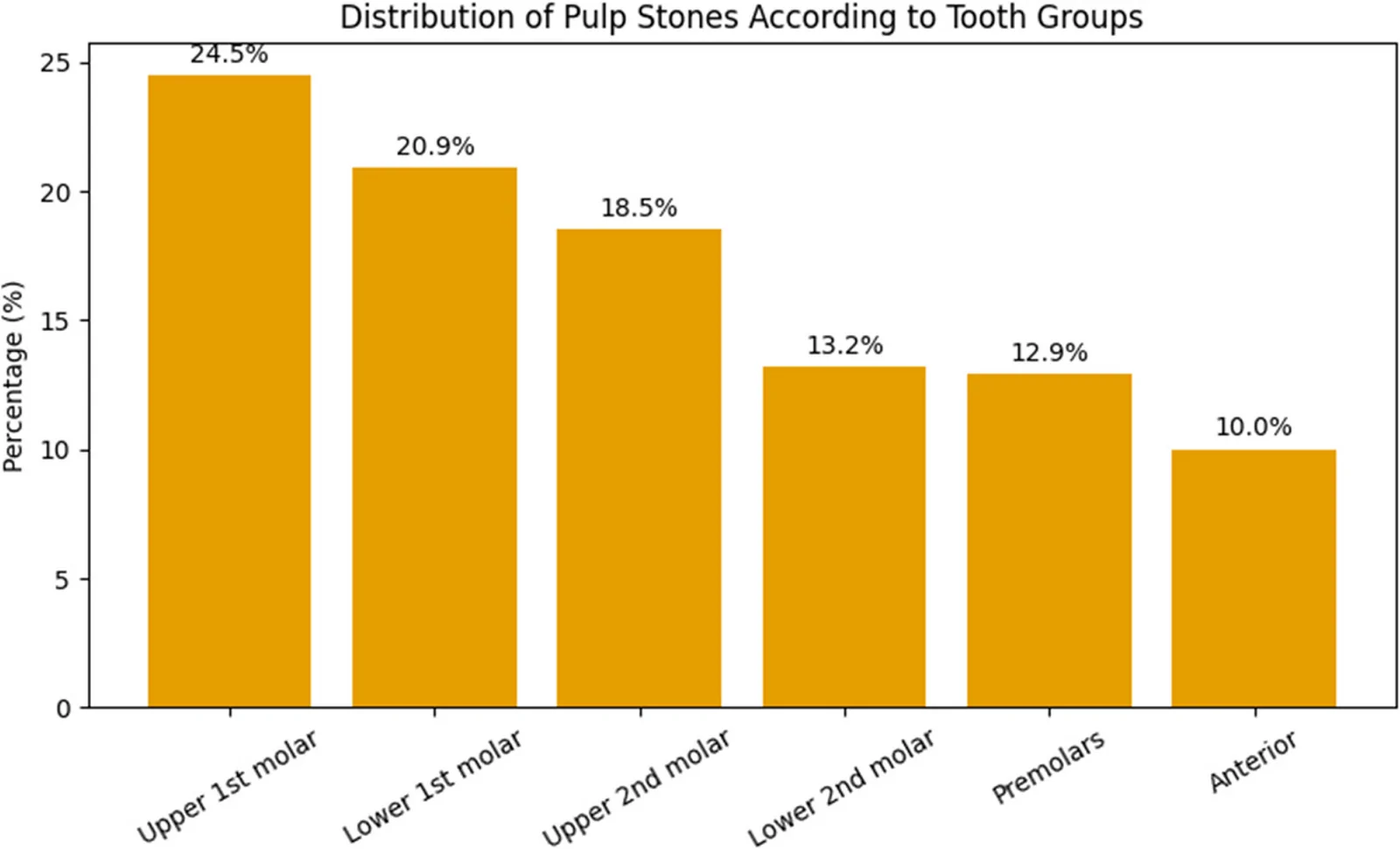
Distribution of pulp stones according to tooth groups

The distribution of radiographically detected pulp stones varied among tooth groups. The highest frequency was observed in upper first molars (*n* = 164; 24.5%), followed by lower first molars (*n* = 140; 20.9%) and upper second molars (*n* = 124; 18.5%). Lower second molars accounted for 13.2% (*n* = 88). Premolars (*n* = 86; 12.9%) and anterior teeth (*n* = 67; 10.0%) showed the lowest frequencies. These findings indicate that radiographically detected pulp stones were observed predominantly in molar teeth, particularly in the upper first molars (Fig. 3).

**Fig. 4:**
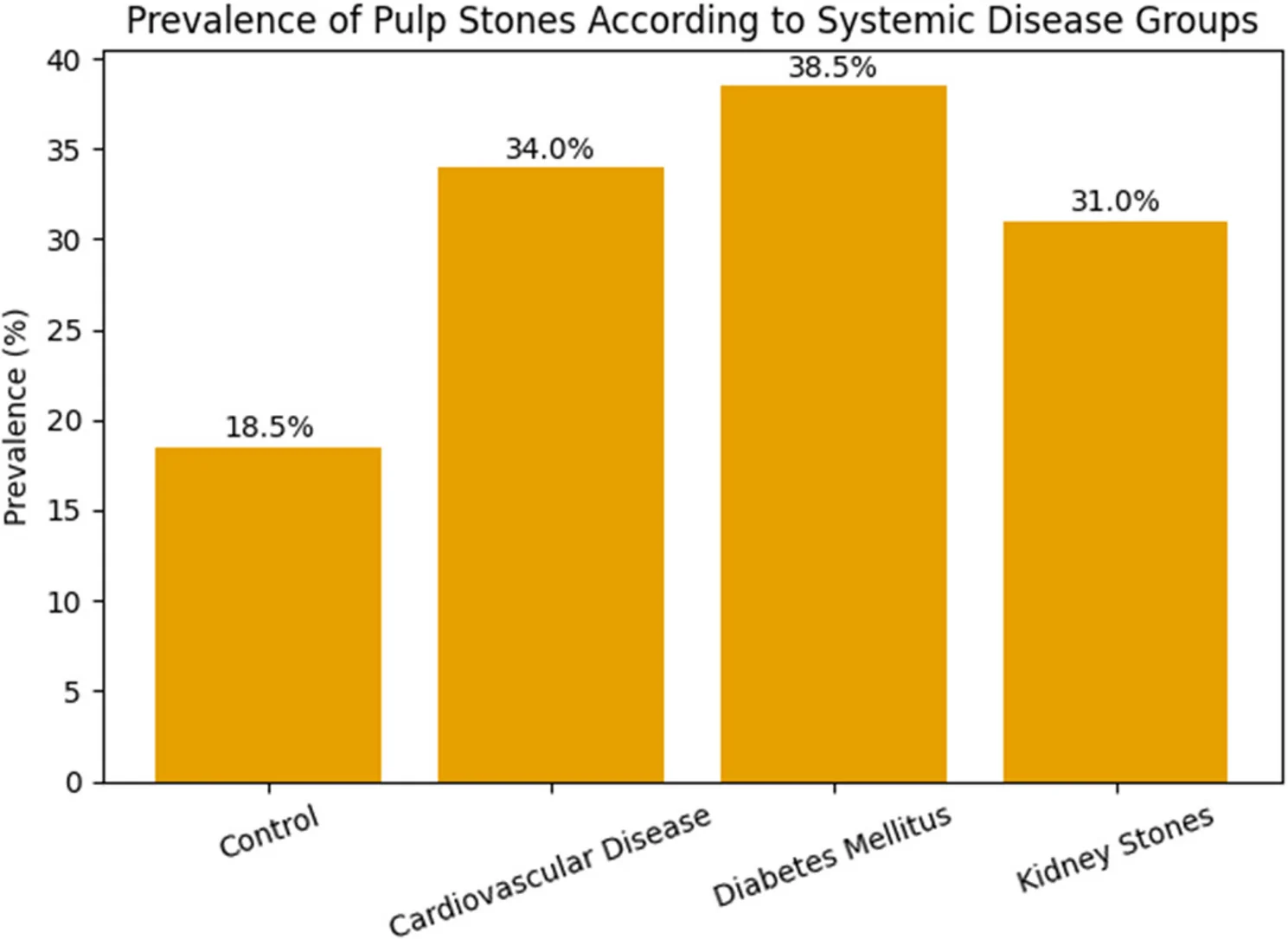
Prevalence of pulp stones according to systemic disease groups

The prevalence of radiographically detected pulp stones differed among the study groups when evaluated according to the number of patients in each group. In the control group (*n* = 400), radiographically detected pulp stones were detected in 74 patients (18.5%). In the cardiovascular disease group (*n* = 200), pulp stones were observed in 68 patients (34.0%). In the DM group (*n* = 200), 77 patients (38.5%) presented with pulp stones, representing the highest prevalence among the groups. In the kidney stones group (*n* = 200), pulp stones were identified in 62 patients (31.0%). These findings indicate that the prevalence of pulp stones was higher in patients with systemic diseases compared with the control group (Fig. 4).

## Discussion

The prevalence of radiographically detected pulp stones has been reported to vary widely among populations. Hamasha and Darwazeh [12] reported a prevalence of 22% in a Jordanian population, while Şener et al. [13] reported 38% in a Turkish population, Bains et al. [14] 41.8% in an Indian population, and Ranjitkar et al. [2] 46.1% in an Australian population. In the present study, the prevalence of pulp stones was found to be 28.1%, which is consistent with the values reported in the literature, although it is lower than some studies and higher than others. These differences may be attributed to variations in imaging modalities, particularly the limited ability of panoramic radiographs to detect small calcifications, as well as differences in sample characteristics and evaluation criteria.

With regard to sex distribution, conflicting results have been reported in the literature. Baghdady et al. [15] reported a higher prevalence in males, whereas Ranjitkar et al. [2] found no significant difference between sexes. In the present study, however, the prevalence of pulp stones was higher in females. This finding is consistent with that of Edds et al. [16]. Although the higher prevalence in females may be related to hormonal and metabolic differences, the influence of sex remains unclear. These inconsistencies suggest that sex-related differences in pulp stone prevalence may be influenced by population characteristics and methodological variations rather than a direct biological effect.

In terms of tooth type and arch distribution, pulp stones were most frequently observed in the maxillary arch and molar teeth, with the highest frequency observed in the maxillary first molars (24.5%). This finding is consistent with studies reporting a higher risk in the maxilla and first molars [15, 17, 18]. The higher frequency of radiographically detected pulp stones in molar teeth may be attributed to their larger pulp chamber volume and richer vascular supply, which may predispose them to calcification. However, some studies have reported a higher frequency of pulp stones in mandibular teeth, and these discrepancies may be due to differences in population characteristics and methodological variations.

When evaluated according to age groups, the prevalence of radiographically detected pulp stones increased up to the 46–60 age group and slightly decreased thereafter. This finding is consistent with the results reported by Gülşahi et al. [19] and Çolak et al. [20]. Age-related changes such as increased secondary dentin formation, reduction in cellular components, and progressive vascular degenerative alterations may explain the higher frequency of pulp calcifications with advancing age. However, some studies have reported contrasting results; for instance, Altındağ and Yüksel [21] reported that pulp calcifications were most frequently observed in younger individuals. These discrepancies may be attributed to differences in sample characteristics and evaluation methods.

Studies investigating the relationship between pulp stones and systemic factors suggest that these calcifications may be associated not only with local factors but also with systemic processes. In a CBCT-based study, the prevalence of pulp stones in unerupted teeth was reported as 24.2% on a jaw basis, and a significant association was found between pulp stone formation and systemic diseases as well as chronic medication use [22]. Similarly, in a large-scale study evaluating digital radiographs of 1030 patients, pulp stones were found to be significantly associated with cardiovascular diseases and DM [23]. In the same study, 86.25% of pulp stones were observed in individuals with systemic diseases, with a higher prevalence particularly in the 46–60 age group. These findings suggest that age-related vascular and degenerative changes may play a role in the development of pulp calcifications.

In the present study, the higher prevalence of pulp stones observed in individuals with systemic diseases is consistent with the existing literature. However, these crude associations changed substantially after adjustment for age and sex, with adjusted estimates moving below the null value. This finding suggests substantial confounding, particularly by age, and limited comparability between the disease and control groups.

The literature indicates that pulp stones are more frequently observed in individuals with cardiovascular diseases [24]. In a CBCT-based study, the frequency of pulp stones was significantly higher in individuals over 50 years of age with cardiovascular disease, with a reported 2.94-fold increased risk [17]. Similarly, other studies have also reported that pulp stones are most commonly observed in patients with cardiovascular conditions among systemic disease groups [25].

In different studies, the prevalence of pulp stones in individuals with ischemic heart disease has been reported to range between 67.3% and 74% [9, 16, 26], and in some cases, it has been reported to be as high as 100% [27]. Additionally, Bernick et al. [28] observed narrowing of the pulpal vascular lumen in individuals with cardiovascular disease, suggesting that vascular alterations may be associated with pulp calcifications. In the present study, the prevalence of pulp stones in individuals with cardiovascular disease was found to be 34%. Although this value is lower than those reported in the literature, the finding may reflect age-related vascular and degenerative changes rather than an independent disease-specific effect.

In the present study, the prevalence of radiographically detected pulp stones in the DM group was found to be 38.5%. Srivastava et al. [17] reported that the risk of pulp stones in diabetic individuals was 1.81 times higher than in healthy subjects. Similarly, other studies have also reported an increased incidence of pulp stones in patients with DM [24, 29, 30]. In the unadjusted analysis, the odds of pulp stone occurrence were higher in the diabetes mellitus (OR = 2.25), cardiovascular disease (OR = 1.89), and kidney stone groups (OR = 1.62) compared with the control group. However, these crude associations changed substantially after adjustment for age and sex, with adjusted estimates moving below the null value. These findings suggest substantial confounding, particularly by age, and indicate that the observed crude associations may largely reflect differences in age distribution between the disease and control groups rather than independent disease-specific effects.

Histopathological studies further support this association. Russell [31] reported angiopathic changes, thickening of the basement membrane, and increased calcifications in the pulp tissue of individuals with long-standing diabetes. In contrast, Bissada and Sharawy [32] did not observe significant vascular differences but reported a higher frequency of amorphous calcified structures in diabetic individuals. Taken together, these findings may indicate that hyperglycemia-related microvascular alterations could contribute to pulpal calcification processes; however, current evidence remains insufficient to support a direct independent association.

The relationship between pulp stones and renal diseases or kidney stones remains controversial in the literature. Ertaş et al. [33] reported a pulp stone prevalence of 72.4% in patients with kidney stones; however, no significant difference was found compared to the control group. Similarly, Mohavvedian et al. [34] reported a prevalence of 49.4% in the kidney stone group and 36.4% in the control group. Although no overall significant association was found, they reported that individuals with pulp stones in three or more teeth had a 5.78-fold increased likelihood of having kidney stones. On the other hand, Näsström et al. [35] reported that narrowing of the pulp chamber was more frequently observed in individuals with renal disease receiving high-dose corticosteroid therapy. Furthermore, a systematic review indicated that due to the dystrophic nature of pulp calcifications, approximately 40% of studies did not find a significant association between pulp stones and kidney stones related to hypercalcemia [1]. In the present study, the prevalence of radiographically detected pulp stones in individuals with kidney stones was 31%. This finding is consistent with the conflicting results reported in the literature and suggests that pulp stones and kidney stones may develop through different pathophysiological mechanisms.

When the methodological reliability of the study was evaluated, a high level of agreement between observers was observed. The obtained kappa values (intra-observer: 0.89; inter-observer: 0.87) indicate good observer consistency and reproducibility. Compared with the similarly high kappa value (κ = 0.94) reported by Aydın et al. [36], the reliability of the present study appears to be consistent with the literature.

Although crude analyses demonstrated higher prevalence rates of radiographically detected pulp stones in individuals with systemic diseases, these associations changed substantially after adjustment for age and sex, with adjusted estimates moving below the null value, suggesting that age-related factors may play a major role in the observed crude associations.

### Strengths and limitations

One of the major strengths of this study is the relatively large sample size and the inclusion of multiple comparative systemic disease groups, which allowed a more direct comparison of different systemic conditions within the same study population. This design provides a more comprehensive evaluation compared to studies focusing on a single systemic disease. In addition, the high intra- and inter-observer agreement values (κ = 0.81–0.89), ranging from good to excellent, indicate good reproducibility and observer consistency in the radiographic assessments.

The present study has several limitations that should be acknowledged. First, the retrospective cross-sectional design does not allow causal inference between radiographically detected pulp stones and systemic diseases. In addition, the retrospective nature of the study limited the availability of important clinical and behavioral variables, including disease duration, glycemic/metabolic control, medication use, smoking habits, periodontal status, bruxism, orthodontic treatment history, restorations, and caries experience, which may have acted as potential confounding factors.

Another important limitation is that pulp stone assessment was based solely on panoramic radiographs. Although panoramic imaging is widely used in epidemiological dental studies because of its accessibility and routine clinical application, it has lower sensitivity than CBCT for detecting small pulpal calcifications and lacks histopathological confirmation. Therefore, the findings should be interpreted as radiographically detected pulp stones rather than definitive pathological diagnoses, and the true prevalence of pulpal calcifications may have been underestimated. In addition, although good inter- and intra-observer agreement was achieved, these findings reflect measurement reliability rather than definitive diagnostic validity.

The findings of the present study should be interpreted cautiously because panoramic radiographs have inherent diagnostic limitations in detecting pulpal calcifications. Due to the two-dimensional nature and limited sensitivity of panoramic imaging, small calcifications may remain undetected, and radiographic differentiation between true pulp stones, diffuse calcifications, and other pulpal radiopacities may not always be possible.

Furthermore, the cardiovascular disease group included heterogeneous conditions such as hypertension and coronary artery disease, which may involve different pathophysiological mechanisms related to pulpal calcifications. Because of subgroup size limitations, separate subgroup analyses could not be performed. Unequal group sizes and possible tooth loss in older or medically compromised individuals may also have influenced the observed associations and radiographic detection rates. Additionally, because multiple teeth from the same patient were evaluated, tooth-level findings should be interpreted cautiously due to potential biological clustering.

Finally, the retrospective single-center design and regional characteristics of the study population may limit the generalizability of the findings to broader populations. Despite these limitations, the present study provides additional epidemiological data regarding the possible association between radiographically detected pulp stones and systemic diseases. Future prospective, multicenter studies using advanced imaging modalities and detailed clinical data are needed to further clarify these associations.

## Conclusion

Within the limitations of this retrospective cross-sectional study, radiographically detected pulp stones were more frequently observed in females and molar teeth, as well as in patients with diabetes mellitus, cardiovascular disease, and kidney stones compared with healthy controls. Although higher crude prevalence rates were observed in systemic disease groups, these associations changed substantially after adjustment for age and sex, with adjusted estimates moving below the null value, suggesting substantial confounding, particularly by age, and limited comparability between the disease and control groups. Therefore, the present findings should be interpreted cautiously and should not be considered evidence of a causal or predictive relationship. In addition, panoramic radiography has limited sensitivity for detecting small pulpal calcifications and does not provide definitive pathological confirmation. Further prospective, multicenter studies using advanced imaging modalities and detailed clinical data are needed to better clarify the nature and clinical significance of these associations.

## Data Availability

The datasets used and/or analyzed during the current study are not publicly available due to patient privacy and institutional restrictions but are available from the corresponding author on reasonable request.

## Abbreviations

CBCT: Cone Beam Computed Tomography
DM: Diabetes Mellitus
